# Comment on Akkaya et al. Posterior Teflon-Felt-Reinforced Coronary Button Anastomosis in a Modified Bentall Procedure: Early Outcomes in a Single-Center Retrospective Study. *J. Clin. Med.* 2026, *15*, 2546

**DOI:** 10.3390/jcm15197555

**Published:** 2026-09-29

**Authors:** Ünsal Vural

**Affiliations:** Department of Cardiovascular Surgery, University of Health Sciences, Dr. Siyami Ersek Thoracic and Cardiovascular Surgery Training and Research Hospital, Istanbul 34668, Türkiye; unsalvural@gmail.com; Tel.: +90-532-200-4351

We read with interest the study by Akkaya et al. describing posterior Teflon-felt reinforcement of coronary button anastomoses during the modified Bentall procedure [1]. The report supports technical feasibility; however, the inference that felt reinforcement improves hemostatic stability and provides additional mechanical support should be interpreted cautiously for several reasons.

The primary issue is causal attribution. Of the nine re-explorations in the conventional group, only two were attributable to coronary button bleeding; the remaining events arose from the proximal suture line or diffuse mediastinal bleeding. None of the three re-explorations in the reinforced group were coronary button-related. Therefore, the intervention-specific comparison is 2/30 versus 0/27, for which a two-sided Fisher’s exact test yields *p* = 0.49. This result neither establishes equivalence nor excludes a clinically relevant benefit. However, it does show that the aggregate 9-versus-3 difference, which combines target-site and unrelated bleeding sources, cannot be attributed specifically to a felt-mediated hemostatic effect.

The cohorts were noncontemporaneous: the conventional cohort spanned January 2021 to January 2024, whereas the reinforced cohort spanned February 2024 to May 2025. Because technique allocation followed calendar time rather than patient- or lesion-specific characteristics, the effect of reinforcement cannot be separated from secular changes in team experience, surgical volume, transfusion practice, or perioperative management. As the reinforced cohort was prospective and the comparator historical, identical outcome ascertainment should be confirmed. The exclusion of one reoperative case only from the reinforced cohort also warrants clarification regarding symmetric eligibility criteria.

Most individual bleeding-related and coronary clinical endpoints did not reach statistical significance. The 24 h drainage volumes were 425 vs. 380 mL (*p* = 0.060), and the Firth-model *p*-values were 0.130 for re-exploration, 0.191 for unplanned coronary bypass, and 0.200 for early mortality; their confidence intervals included unity. Significant differences were mainly confined to blood-product utilization. However, transfusion thresholds, preoperative antiplatelet or anticoagulant therapy, coagulation results, antifibrinolytic use, and viscoelastic-guided protocols were not reported. Moreover, although postoperative bleeding was defined as drainage exceeding 1.5 mL/kg/h for six consecutive hours, the number of patients meeting this prespecified criterion was not presented. These details are necessary before lower transfusion requirements can be attributed specifically to coronary felt reinforcement.

A further statistical clarification is needed: for the aggregate re-exploration comparison of 9/30 versus 3/27, the manuscript reports Fisher’s exact *p* = 0.076, whereas a standard two-sided Fisher’s exact test yields approximately 0.109. Specification of the exact alternative hypothesis and any correction applied would improve reproducibility. Coronary safety and patency were also not objectively assessed by routine postoperative coronary computed tomography angiography or invasive angiography. Two reinforced patients required supplementary sutures for minor kinking and an uneven right coronary button configuration. Thus, feasibility is shown, but anatomical superiority, durable patency, and longer-term safety are not established.

Finally, benefit in fragile tissue remains unsupported because tissue quality was not analyzed and acute dissection was excluded. Overall, routine reinforcement requires confirmation with contemporaneous controls, standardized hemostatic/transfusion protocols, and objective coronary imaging.

## Data Availability

No new data were created or analyzed in this work. Data sharing is not applicable.
